# Unravelling diagnostic clusters and immune landscapes of cuproptosis patterns in intervertebral disc degeneration through dry and wet experiments

**DOI:** 10.18632/aging.205449

**Published:** 2023-12-29

**Authors:** Peng Zhang, Jiahui He, Yanchi Gan, Qi Shang, Honglin Chen, Wenhua Zhao, Jianchao Cui, Gengyang Shen, Yuwei Li, Xiaobing Jiang, Guangye Zhu, Hui Ren

**Affiliations:** 1 Guangzhou University of Chinese Medicine, Guangzhou 510405, China; 2 The Affiliated TCM Hospital of Guangzhou Medical University, Guangzhou 510130, China; 3 The Second Affiliated Hospital of Guangzhou Medical University, Guangzhou 510260, China; 4 The First Affiliated Hospital of Guangzhou University of Chinese Medicine, Guangzhou 510405, China; 5 Suzhou TCM Hospital Affiliated to Nanjing University of Chinese Medicine, Suzhou 215007, China

**Keywords:** intervertebral disc degeneration, cuproptosis biomarkers, subtype classification, immune microenvironment infiltration, experimental validation

## Abstract

Cuproptosis is a manner of mitochondrial cell death induced by copper. However, cuproptosis modulators’ molecular processes in intervertebral disc degeneration (IDD) are still unclear. To better understand the processes of cuproptosis regulators in IDD, a thorough analysis of cuproptosis regulators in the diagnostic biomarkers and subtype determination of IDD was conducted. Then we collected clinical IDD samples and successfully established IDD model *in vivo* and *in vitro*, and carried out real-time quantitative polymerase chain reaction (RT-qPCR) validation of significant cuproptosis modulators. Totally we identified 8 crucial cuproptosis regulators in the present research. Using a random forest model, we isolated 8 diagnostic cuproptosis modulators for the prediction of IDD risk. Then, based on our following decision curve analysis, we selected the five diagnostic cuproptosis regulators with importance scores greater than two and built a nomogram model. Using a consensus clustering method, we divided IDD patients into two cuproptosis clusters (clusterA and clusterB) based on the important cuproptosis regulators. Additionally, each sample’s cuproptosis value was evaluated using principal component analysis in order to quantify the cuproptosis clusters. Patients in clusterB had higher cuproptosis scores than patients in clusterA. Moreover, we found that clusterB was involved in the immunity of natural killer cell, while clusterA was related to activated CD4 T cell, activated B cell, etc. Notably, cuproptosis modulators detected by RT-qPCR showed generally consistent expression levels with the bioinformatics results. To sum up, cuproptosis modulators play a crucial role in the pathogenic process of IDD, providing biomarkers and immunotherapeutic approaches for IDD.

## INTRODUCTION

With the aging of society, the prevalence of chronic inflammatory diseases, including degenerative disc disease, is increasing [1]. Intervertebral disc degeneration (IDD) is closely associated with age, load-bearing, trauma, genetics, inflammation, and tissue injury induced by oxidative stress [2]. IDD is an important factor in lower back pain, which is a common clinical condition that causes long-term pain and, potentially, the inability to work; it seriously affects life quality, and increases a major social and economic strain on families and economy [3, 4]. Existing drugs serving as suggested treatments for the management of IDD don’t focus on its pathogenesis but the symptoms, and some side effects are triggered for long-term use causing frequent recurrence and gradual aggravation [5]. Therefore, clinical management of IDD is still lacking. It is indispensable and critical to find new prognostic factors and therapeutic targets for IDD so as to early recognize individuals who are at an elevated risk of IDD. There is growing proof from the numerous advancements in IDD study that it is a complex, highly heterogeneous disorder that includes genetic changes [6]. It follows that early detection and effective avoidance of high-risk individuals from a genetic viewpoint will have a significant impact on the epidemiological management of IDD.

Cuproptosis is a copper-triggered way of mitochondrial cell death depending on mitochondrial respiration, which happens through copper combining directly with the lipoylated components of the tricarboxylic acid (TCA) cycle, and then results in the aggregation of lipoylated proteins and the subsequent loss of iron-sulphur cluster proteins, resulting in proteotoxic stress and ultimately cell death [7]. As a distinct cell death mechanism, cuproptosis needs numerous copper-regulatory proteins including FDX1, DBT, GCSH, LIPT1, DLST, LIAS, DLD, DLAT, ATP7A, ATP7B, MTF1, GLS, PDHA1, PDHB, SLC31A1, and CDKN2A to cooperate together [7, 8]. Abnormalities in copper metabolism can trigger multiple disorders such as obesity [9], glioblastoma [10], acute myeloid leukaemia [11], type 2 diabetes [12], and various malignancies [13]. With the further study on copper, researchers have found that the concentration of copper element shows significant differences between the tissues of IDD and normal controls [14], and copper could result in severe disc degeneration [15]. Therefore, we hypothesize that cuproptosis may influence the pathogenesis of IDD by modifying the transcript level of genes related to cuproptosis. To date, there exist no research on the roles of cuproptosis modulators in IDD.

**Figure 1:**
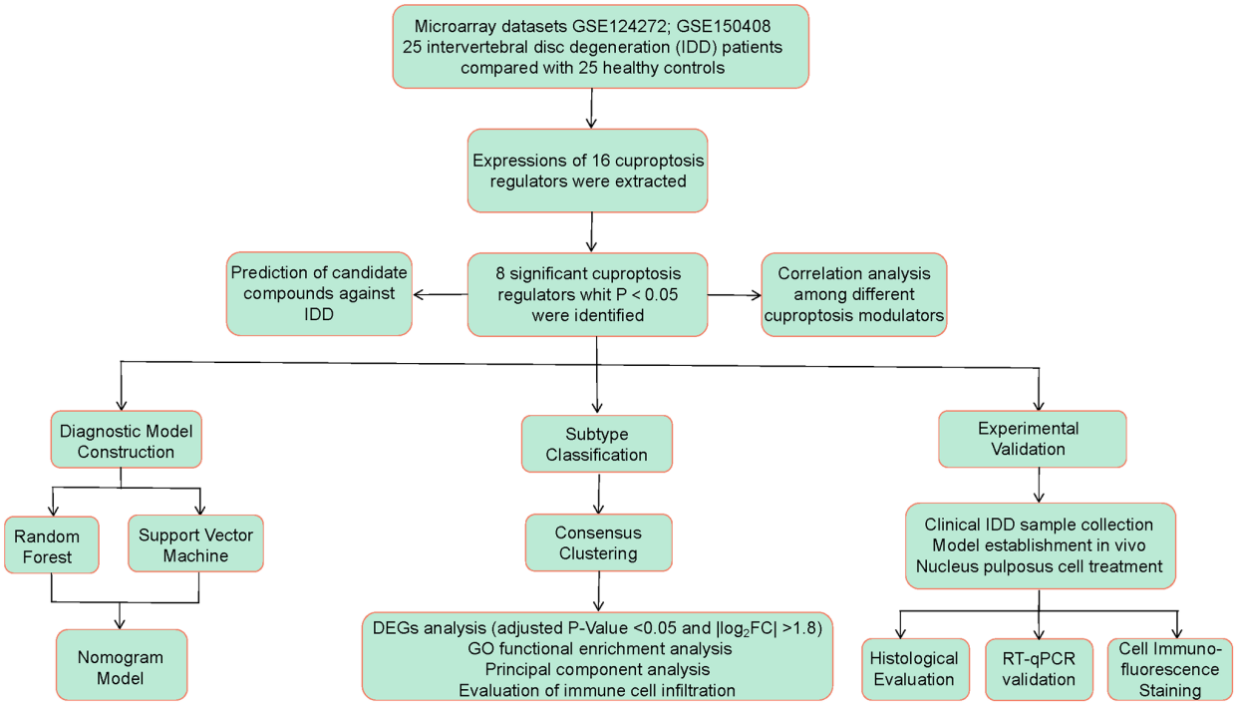
Flow chart of the study design.

On the basis of the GSE124272 [16] and GSE150408 [17] datasets, we conducted a thorough assessment of the functions of cuproptosis regulators in the identification of IDD subtypes and diagnostic biomarkers in this research. Based on eight important cuproptosis modulators, including LIPT1, GCSH, DLST, DLAT, PDHB, ATP7A, ATP7B, and MTF1, we created an IDD susceptibility prediction gene model. We discovered that the model was helpful for offering patients predictive value clinically. These cuproptosis regulators were further confirmed by our RT-qPCR assessments, which showed compatible expression levels with the bioinformatics findings. Thereafter, we identified two different cuproptosis patterns that were closely correlated with activated CD4 T cell, activated B cell, activated CD8 T cell, natural killer cell, immature B cell, and T follicular helper cell immunity, demonstrating that cuproptosis patterns may be employed to recognize IDD and offer following therapy plans. Figure 1 displayed the flowchart of study design and process.

## MATERIALS AND METHODS

### Bioinformatics analysis

#### Retrieval of IDD samples

Using the GEO database (http://www.ncbi.nlm.nih.gov/geo/), we obtained whole blood samples of IDD patients. “Intervertebral Disc Degeneration”, “Whole Blood”, “Gene expression”, “Microarray” were the search phrases, and the datasets were chosen according to the eligibility principles: the dataset has at least 10 samples, containing at least 5 cases respectively in the control and IDD groups, which also provides both raw data and series matrix file available to download. Finally, the datasets, GSE124272 [16] and GSE150408 [17] were selected, which fully satisfied our requirements. We selected 8 cases of control group and 8 cases of IDD group from the dataset of GSE124272 as well as 17 cases of IDD and 17 controls in GSE150408 dataset.

#### Data collection

To convert microarray probes to symbols, we utilized the annotated R package (v4.1.2) downloaded from Bioconductor (http://bioconductor.org/). Following data preparation, the two datasets were merged via removing bath effects in virtue of SVA package using ComBat function. Thereafter, we obtained the final dataset containing 25 controls and 25 IDD cases. A difference analysis of control and IDD cases was conducted using Limma package to determine differential cuproptosis regulators. Significant cuproptosis regulators were selected based on the following screening criteria: *P*-Value < 0.05 and |log_2_ fold change (FC)| >0 [18]. We then analyzed the enrichment results of GO and KEGG using the R package “clusterProfiler” with the adjusted *P*-value threshold of 0.05 so as to investigate the possible mechanism of the cuproptosis modulators in IDD.

#### Model establishment

To analyze the occurrence of IDD, we constructed random forest (RF) and support vector machine (SVM) models as training models, which were determined by “Reverse cumulative distribution of residual”, and “Boxplots of residual”. We built an RF model using the R package of “RandomForest” to select putative cuproptosis regulators with importance score (Mean Decrease Gini) >1. The number n in SVM model represented the number of cuproptosis regulators and every data dot was shown as a dot in an n-dimensional space. We then chose an ideal hyperplane that differentiated between the two groups of control and IDD [19]. Thereafter, the R package of “rms” was utilized to construct a nomogram model to evaluate the prevalence of IDD patients based on selective candidate cuproptosis regulators. Calibration curves were employed to determine how well the prediction values matched reality. Decision curve analysis (DCA) was performed to establish a clinical impact curve and determine if decisions based on the model benefited patients [20].

#### Subtype identification

Using consensus clustering relying on resampling algorithm, each member and its subcluster number were identified, validating the rationality of the clusters [20]. Based on significant cuproptosis regulators, the R package of “ConsensusClusterPlus” was utilized to identify different cuproptosis patterns using a consensus clustering approach [21].

#### GO enrichment analysis of differentially expressed genes between different cuproptosis clusters

Using the Limma package, we screened differentially expressed genes (DEGs) between different cuproptosis clusters according to a threshold of adjusted *P*-Value <0.05 and |log_2_FC| >1.8. Thereafter, GO analysis was conducted using the R package of “clusterProfiler” as to elucidate the underlying mechanism of DEGs in the process of IDD [22].

#### Cuproptosis score assessment

The cuproptosis score for each sample was calculated using principal component analysis (PCA), aiming to quantify the cuproptosis patterns. The cuproptosis score system was established according to the following formula: cuproptosis score = PC1_i_, where PC1 represented principal component 1, and i represented cuproptosis gene expression [23].

#### Immune cell infiltration assessment

The quantity of immune cell infiltration in IDD group samples was assessed using single sample gene set enrichment analysis (ssGSEA). To begin, ssGSEA was used to sequence the gene expression levels in the samples in order to generate a gene expression ranking. Thereafter, the expression levels of the significant cuproptosis modulators were then summed in the input dataset. We obtained the amount of immune cells in each sample based on the above analysis [24].

#### Prediction of candidate compounds against IDD

Protein-compound interaction prediction or candidate compound identification is one of the important elements of this study. Based on the significant cuproptosis genes, candidate compounds were screened via Enrichr [25] by searching DSigDB database [26], which has 22527 gene sets according to its accessible way through Enrichr under the Diseases/Drugs function [27].

### Experimental validation

#### IDD model establishment

This experimental study was approved by the Ethics Committee of The First Affiliated Hospital of Guangzhou University of Chinese Medicine (No. TCMF1-2021026). All the experiments were performed in accordance with relevant ethical guidelines and regulations. Above study is reported in accordance with ARRIVE guidelines. We bought randomly divided 3-month-old wild type C57BL/6 mice into two groups: control (Con, *n* = 6), and IDD (*n* = 6). The animals were kept in a sterile environment with consistent light (12 hours/day), temperature (21~26°C), and humidity (41~70%) conditions, and were provided with adequate water and food. After 24 months, we obtained an IDD model of naturally aging mice according to a previous report [28].

#### Histological evaluation

Serial sections from L4/5 intervertebral discs of 5 μm thickness were taken from the midsagittal region for histological analysis. We performed hematoxylin-eosin (HE) and safranin O/green staining of the slices to observe histological changes in the intervertebral disc tissues from the different groups.

#### Nucleus pulposus tissue acquisition

The human nucleus pulposus (NP) tissues were collected from patients undergoing spine operations at our hospital. The relatively normal human lumbar NP tissues without severe IDD were collected from 3 patients (grade II), while the degenerated NP tissues were collected from 3 patients (grade V). The degenerated degree of the disc was graded by the Pfirrmann classification [29]. All study procedures were approved by the Ethics Committee of The First Affiliated Hospital of Guangzhou University of Chinese Medicine (No. JY2022-122). The informed consents were obtained from patients. All methods were carried out in accordance with the Declaration of Helsinki for human participants.

Mice from the two groups were sacrificed after anesthesia, and then soaked in 75% ethanol for 5 min. Mice were placed in a prone position on an ultra-clean table with skin cut along the midline of the back, muscle tissue stripped off, and spine exposed. After the removal of surrounding tissues including muscle, spinal cord and nerve tissue, the vertebral body and posterior complex structure were separated. The L4/5 vertebrae-disc structure was soaked in sterile HBSS liquid and then nucleus pulposus tissue was isolated according to the morphological difference of the cells with sterile microforceps and scalpels under microscope [30].

#### Mouse NP cell culture and treatment

We obtained nucleus pulposus tissues from mice via digestion of 0.25% trypsin (Gibco, Waltham, MA, USA) for half an hour and 0.25% type II collagenase (Sigma, St. Louis, MO, USA) for 8 hours at 37°C. After centrifugation, NP cells were harvested and incubated in complete DMEM/F12 medium with 1% penicillin-streptomycin and 15% fetal bovine serum, under hypoxic conditions (5% CO_2_ in a humidified incubator at 37°C). Second-generation NP cells were collected for subsequent experiments to prevent their differentiation [30, 31].

#### Cell counting Kit-8 assay

The mouse NP cells were treated with tert-butyl hydrogen peroxide (TBHP, Macklin, Shanghai, China) in the concentration gradients of 0, 50, 100, 150, 200, and 250 μmol/L for 2, 4, and 6 h to establish the IDD cell model *in vitro*. Then, Cell Counting Kit-8 (GK10001, GlpBio, Montclair, CA, USA) assay was conducted to detect cell viability in different groups.

#### Cell immunofluorescence staining

Mouse NP cells were cultured in 24-well plates (4 × 10^4^ cells/well) and fixed for 15–20 min with 4% paraformaldehyde. After washing with PBS containing 0.1% Tween-20 (PBST), the samples were incubated with 0.2% Triton X-100 for 15 min and then blocked with QuickBlock™ Blocking Buffer for Immunol Staining (P0260) for 30–60 min. ACAN (13880-1-AP) was purchased from Proteintech (Wuhan, China). The cells were treated with primary antibodies against ACAN (1:100) overnight at 4°C. Secondary antibodies Goat Anti-Mouse IgG H&L (Alexa Fluor^®^ 488) (ab150113) coupled with fluorescein isothiocyanate were then incubated at 37°C for 1 h. Fluorescence images were obtained by fluorescence microscopy (Leica Microsystems, Inc., Buffalo Grove, IL, USA).

#### RNA extraction and RT-qPCR validation

Human NP tissues as well as mouse NP tissues and cells in this study were collected, and then total RNA from the samples was extracted by TRIzol reagent. Subsequently, a cDNA synthesis kit (Takara Inc., Shiga, Japan) was used to finish retrotranscription of 1 μg total RNA. 20 μL SYBR Green qPCR SuperMix (Takara Inc.) was adopted for detecting cuproptosis gene cDNAs by RT-qPCR machine (QuantStudio 3 and 5 Real-Time PCR Systems). The final gene amplification was performed under the thermal cycling conditions: 95°C for 30 s, 40 cycles of 95°C for 5 s, and a final step of 60°C for 30 s. Quantitative analysis was conducted using the 2^ΔΔCT^ method for calculation of the relative expression of each gene. The gene-related detection primers of cuproptosis modulators were supplied by Shanghai Sangon Biotechnology Co., Ltd., (Shanghai, China), as shown in Supplementary Table 1.

### Statistical analysis

Linear regression analysis was used to evaluate the correlations among the significant cuproptosis genes. In bioinformatics analysis, Kruskal-Wallis tests were used to calculate the differences between groups, while Student’s *t*-tests were used for RT-qPCR analysis. All parametric data were estimated using two-tailed tests, with *P*-value of 0.05 being deemed statistically significant. The mean ± standard deviations were used to describe all results.

### Availability of data and materials

All the data from the present study can be obtained from the first author on reasonable request. The datasets generated and analyzed during the current study are available in the Gene Expression Omnibus (GEO) repository, (GSE124272 and GSE150408).

## RESULTS

### Identification of the 16 cuproptosis modulators in IDD

**Figure 2:**
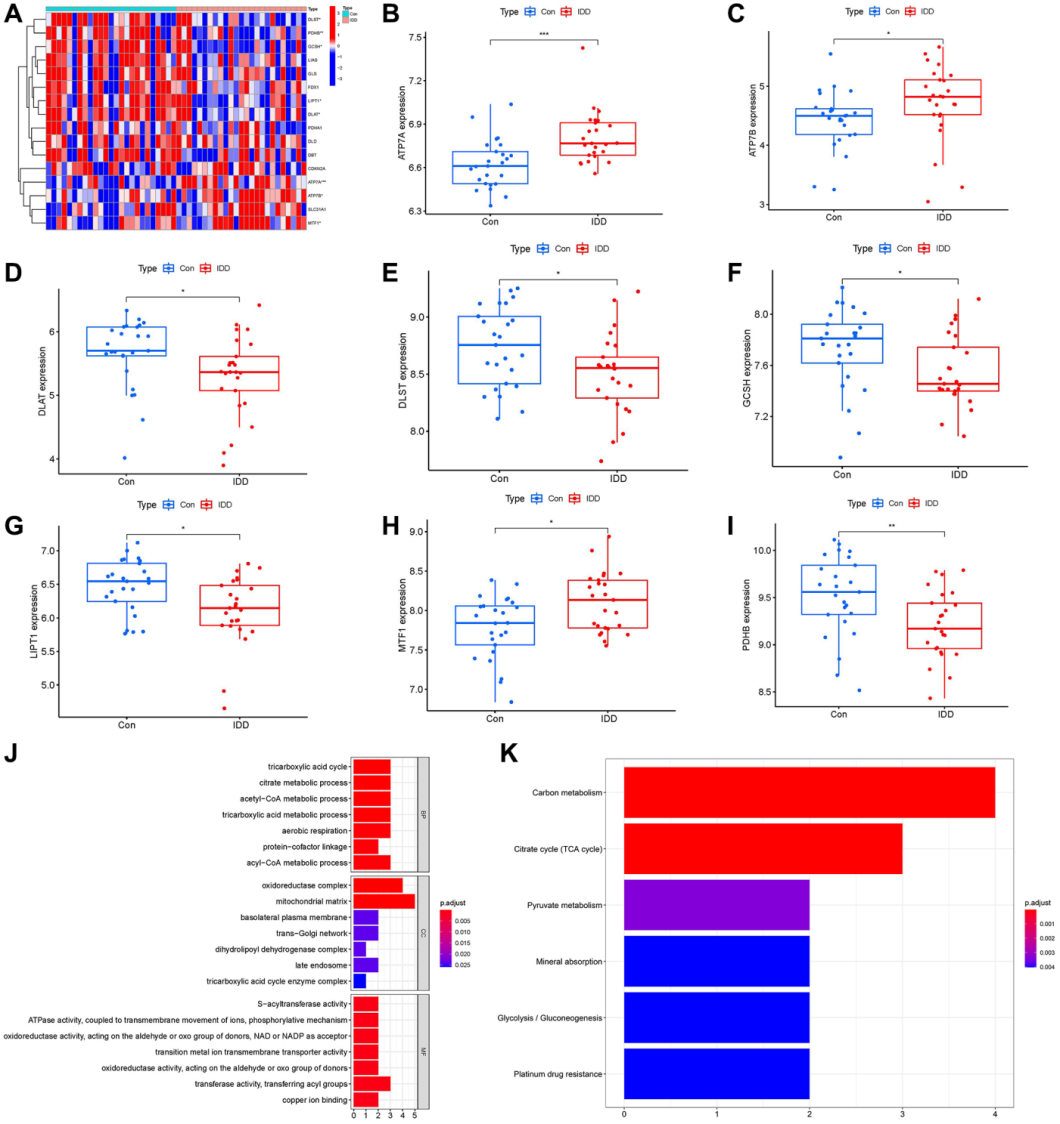
**Identification of the 16 cuproptosis modulators in IDD.** (**A**) Expression heat map of the 16 cuproptosis modulators in controls and IDD cases. (**B**–**I**) Differential expression boxplots of 8 significant cuproptosis modulators identified between controls and IDD cases. (**J**, **K**) GO and KEGG enrichment analysis based on the 8 significant cuproptosis modulators. ^*^p < 0.05, ^**^p < 0.01, and ^***^p < 0.001.

A total of 16 cuproptosis regulators (FDX1, DLD, LIAS, LIPT1, DLST, DLAT, DBT, GCSH, PDHA1, ATP7A, ATP7B, PDHB, SLC31A1, MTF1, GLS, CDKN2A) were screened according to difference analysis between the control and IDD groups. We finally filtrated 8 vital cuproptosis modulators (LIPT1, DLAT, PDHB, GCSH, DLST, ATP7A, ATP7B and MTF1), which were plotted in a heat map (Figure 2A). We noticed that the expressions of ATP7A, ATP7B and MTF1 were increased in IDD cases compared to controls, while the other significant cuproptosis regulators exhibited the opposite results (Figure 2B–2I). Moreover, we carried out GO and KEGG enrichment analyses to excavate the role of these vital cuproptosis modulators in IDD (Figure 2J, 2K). We observed that GO enrichment results including biological process (tricarboxylic acid metabolic process), cellular component (mitochondrial matrix), molecular function (ATPase activity) were the mainly enriched entries. Notably, KEGG enrichment analysis showed that Citrate cycle (TCA cycle) was one of the mainly enriched pathways. The detailed information of GO and KEGG enrichment analysis was shown in Supplementary Tables 2, 3.

### Correlation among cuproptosis regulators in IDD

**Figure 3:**
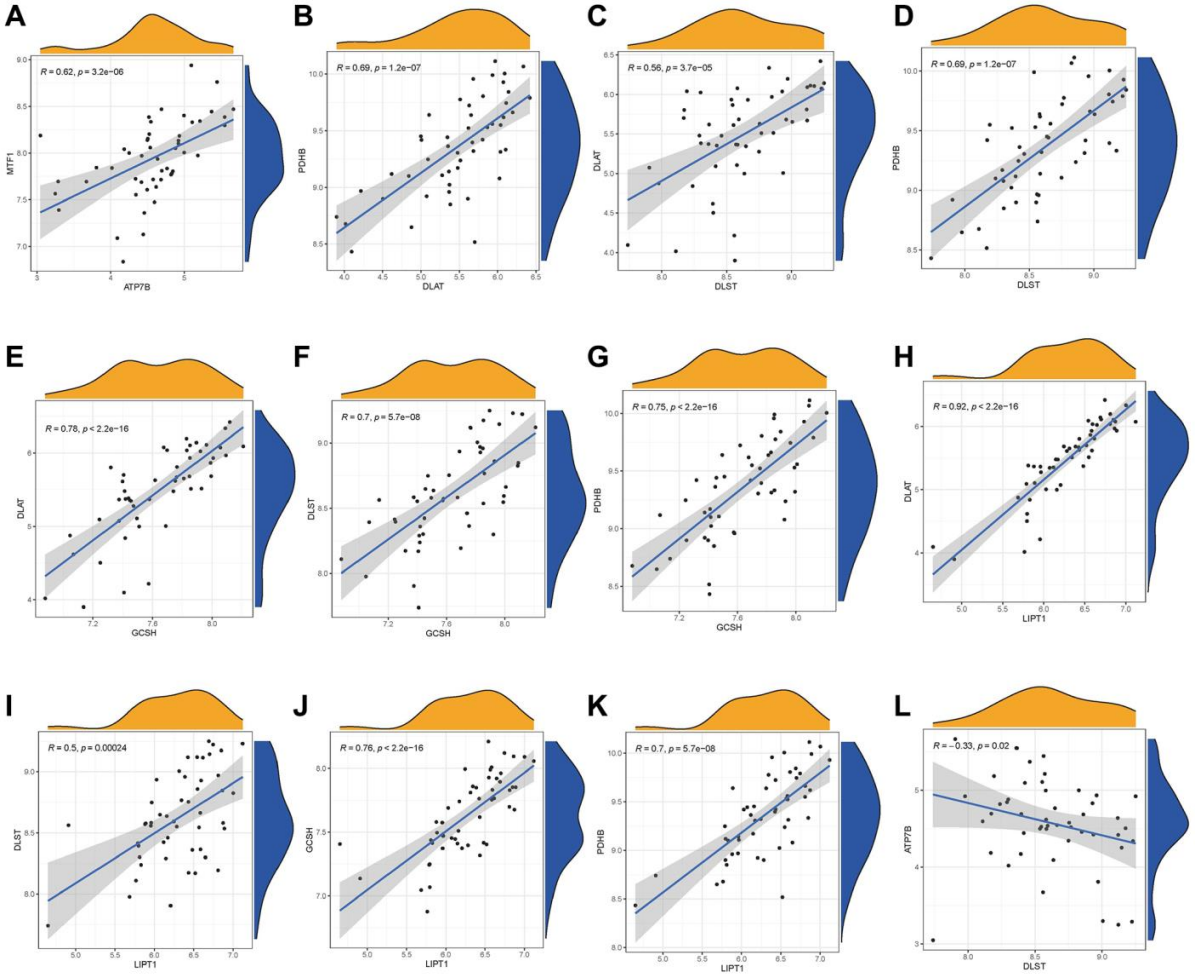
**Correlation among cuproptosis modulators in IDD.** There existed significantly positive correlations in the gene expression levels of MTF1-ATP7B, PDHB-DLAT, PDHB-DLST, DLAT-DLST, DLAT-GCSH, DLST-GCSH, PDHB-GCSH, DLAT-LIPT1, DLST-LIPT1, GCSH-LIPT1, and PDHB-LIPT1 in IDD cases (**A**–**K**), while the gene expression levels of ATP7B-DLST in IDD cases exhibited significantly negative correlation (**L**).

We utilized linear regression analyses to investigate whether gene expression levels of cuproptosis modulators in IDD exhibit correlation with each other. There existed significantly positive correlations in the gene expression levels of MTF1-ATP7B, PDHB-DLAT, PDHB-DLST, DLAT-DLST, DLAT-GCSH, DLST-GCSH, PDHB-GCSH, DLAT-LIPT1, DLST-LIPT1, GCSH-LIPT1, and PDHB-LIPT1 in IDD cases (Figure 3A–3K), while the gene expression levels of ATP7B-DLST in IDD cases exhibited significantly negative correlation (Figure 3L). Thus, we demonstrated different correlations between different cuproptosis modulators in IDD.

### Establishment of the RF and SVM models

**Figure 4:**
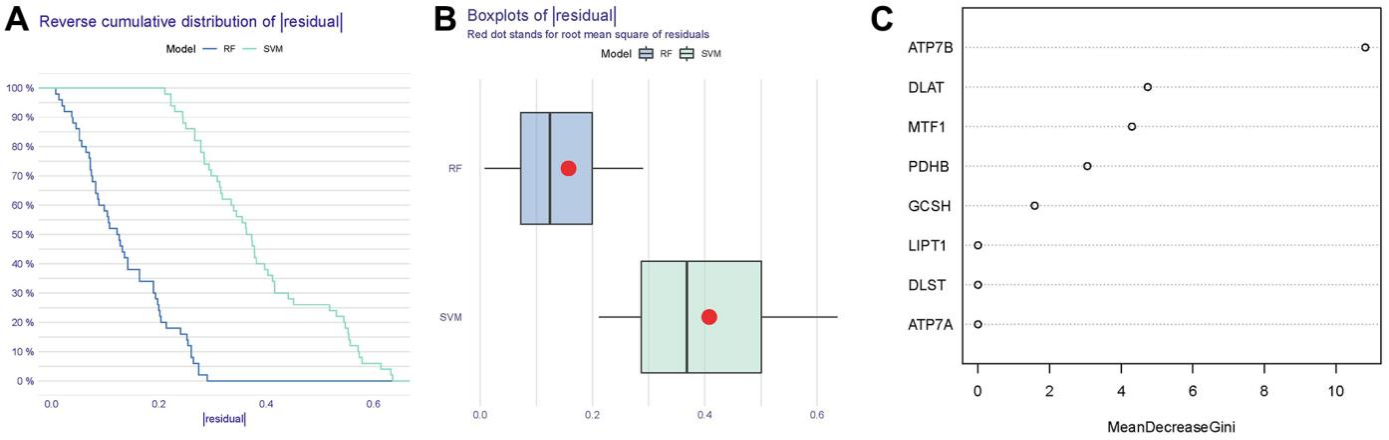
**Establishment of the RF and SVM models.** (**A**) Reverse cumulative distribution of residual was constructed to display the residual distribution of RF and SVM models. (**B**) Boxplots of residual were constructed to display the residual distribution of RF and SVM models. (**C**) The importance of the 8 cuproptosis modulators on the basis of the RF model.

As shown in Figure 4A, 4B, reverse cumulative distribution of residual and boxplots of residual confirmed that the RF model had the smallest residuals. The majority of samples from the RF model had smaller residuals, which suggested that the RF model was superior to the SVM model. Thereafter, we determined the RF model to be the most suitable model for the prediction of IDD occurrence. Finally, we presented these 8 significant cuproptosis regulators after ranking them in order of importance score (Mean Decrease Gini) and selected 5 cuproptosis regulators (ATP7B, DLAT, MTF1, PDHB, GCSH) with importance score >1 as the candidate genes (Figure 4C).

### Nomogram model construction

**Figure 5:**
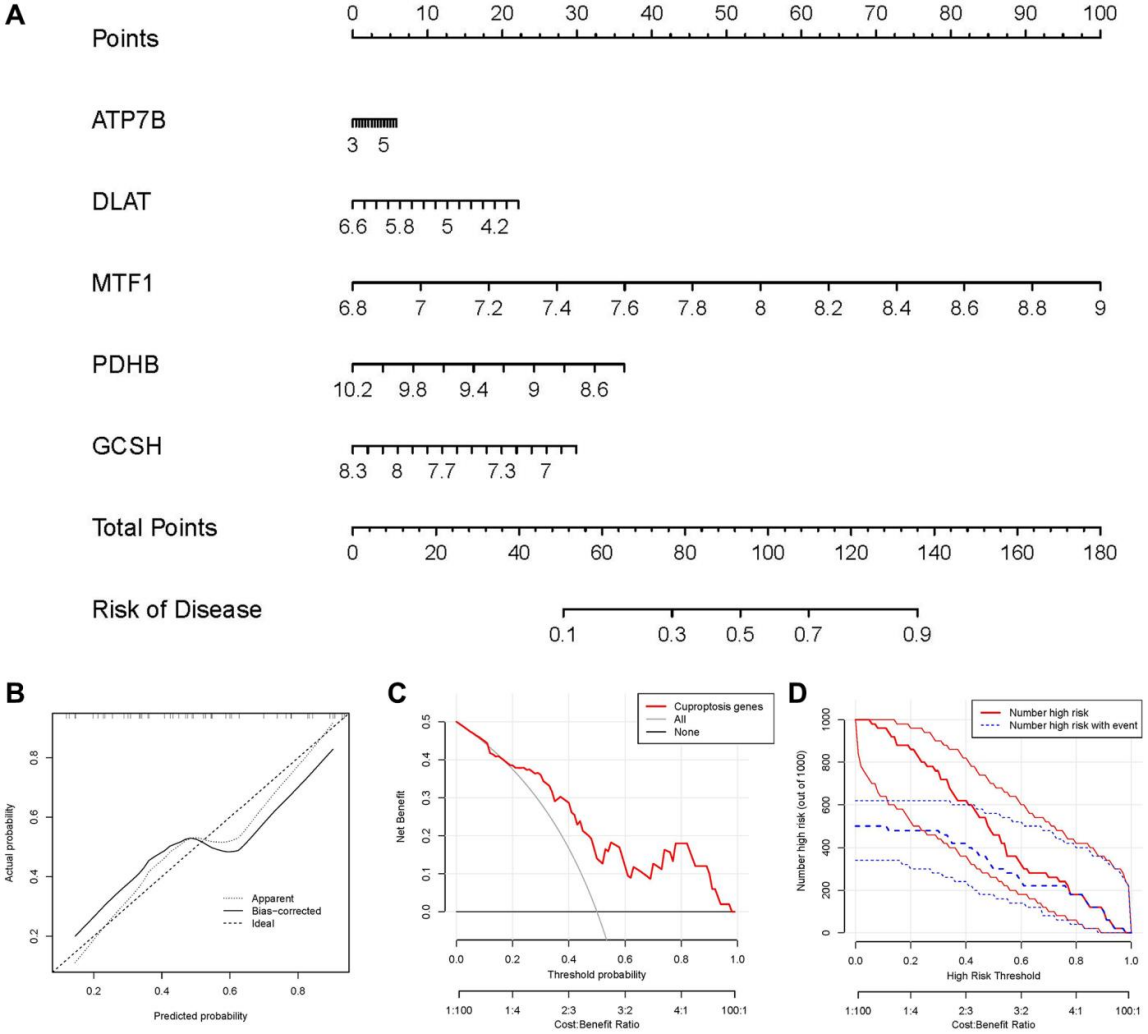
**Establishment of the nomogram model.** (**A**) The nomogram model was established on the basis of the 5 candidate cuproptosis modulators. (**B**) The calibration curve was utilized to evaluate the predictive accuracy of the nomogram model. (**C**) Decisions on the basis of this nomogram model may be beneficial to IDD patients. (**D**) The clinical impact curve was used to assess clinical impact of the nomogram model.

For the purpose of predicting the prevalence of IDD patients, we constructed a nomogram model of the five candidate cuproptosis regulators using the “rms” package in R (Figure 5A). We found that the nomogram model demonstrated high prediction accuracy based on calibration curves (Figure 5B). From 0 to 1, the red line in the DCA curve continued to be higher than the gray and black lines, indicating that IDD patients may benefit from judgments made using the nomogram model (Figure 5C). Additionally, we observed that the predictive capacity of the nomogram model was remarkable on the basis of the clinical impact curve (Figure 5D).

### Recognition of two different cuproptosis clusters

**Figure 6:**
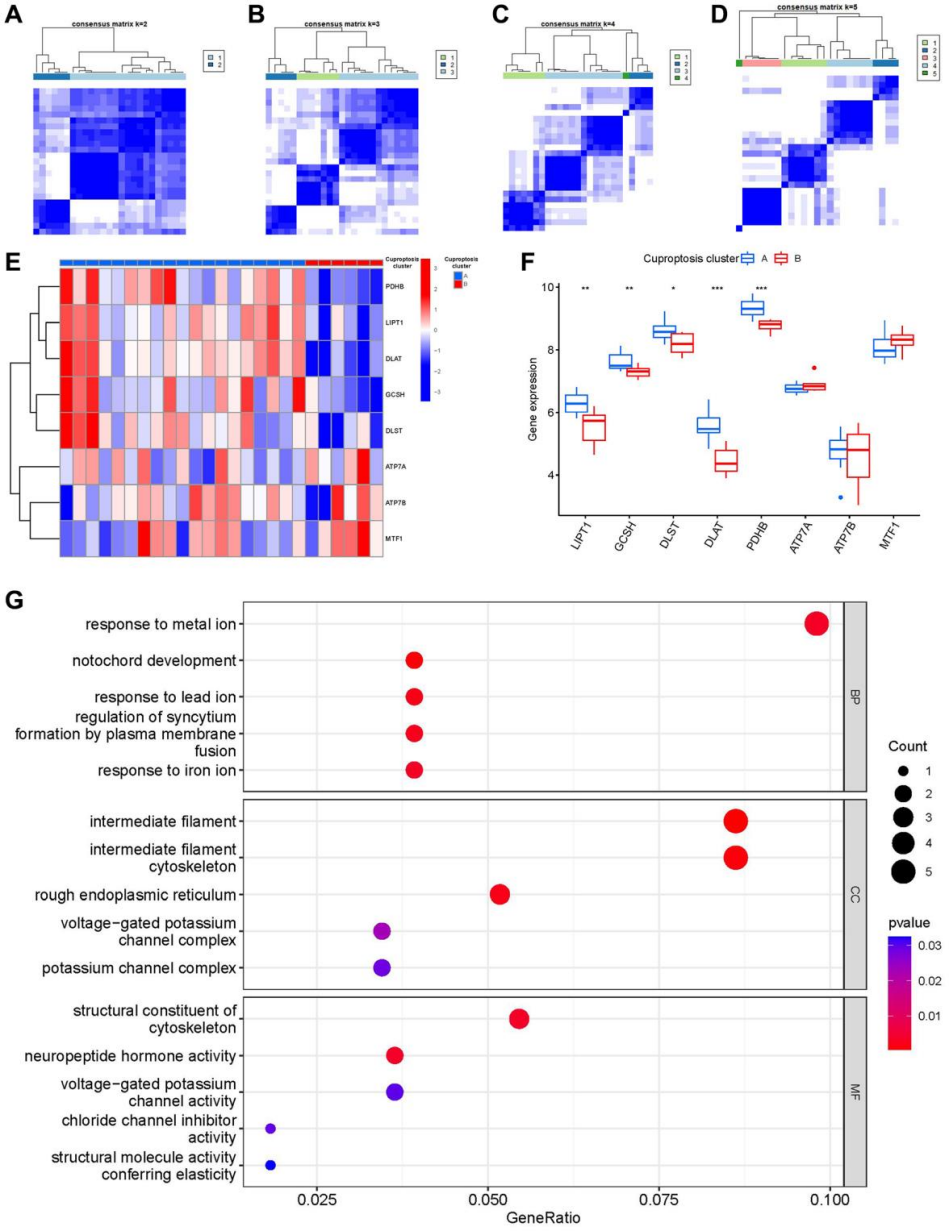
**Consensus clustering of the 8 significant cuproptosis modulators in IDD.** (**A**–**D**) Consensus matrices of the 8 significant cuproptosis modulators for k = 2–5. (**E**) Expression heat map of the 8 significant cuproptosis modulators in clusterA and clusterB. (**F**) Differential expression boxplot of the 8 significant cuproptosis modulators in clusterA and clusterB. (**G**) GO analysis that explores the potential mechanism underlying the effect of the 163 cuproptosis-related DEGs on the occurrence and development of IDD. ^*^p < 0.05, ^**^p < 0.01, and ^***^p < 0.001.

Using the R package of “ConsensusClusterPlus”, we discovered two cuproptosis patterns (clusterA and clusterB) based on the 8 important cuproptosis regulators (Figure 6A–6D). ClusterA contained 19 samples, whereas clusterB had 6 samples. Thereafter, the differential expression levels of the 8 important cuproptosis modulators between the two clusters were clearly demonstrated in the heat map and boxplot. We noticed that the expression levels of LIPT1, GCSH, DLST, DLAT and PDHB in clusterA were higher than those in clusterB (Figure 6E, 6F). We screened totally 163 cuproptosis-associated DEGs between the two cuproptosis patterns, and we carried out GO enrichment analysis to excavate the role of these DEGs in IDD (Figure 6G). The detailed information of GO enrichment analysis was shown in Supplementary Table 4. Specifically, biological progresses included response to metal ion, notochord development, response to lead ion, regulation of syncytium formation by plasma membrane fusion and response to iron ion, etc. Cellular components included intermediate filament, intermediate filament cytoskeleton, rough endoplasmic reticulum, etc. Molecular functions included structural constituent of cytoskeleton, neuropeptide hormone activity, chloride channel inhibitor activity, structural molecule activity and conferring elasticity, etc.

**Figure 7:**
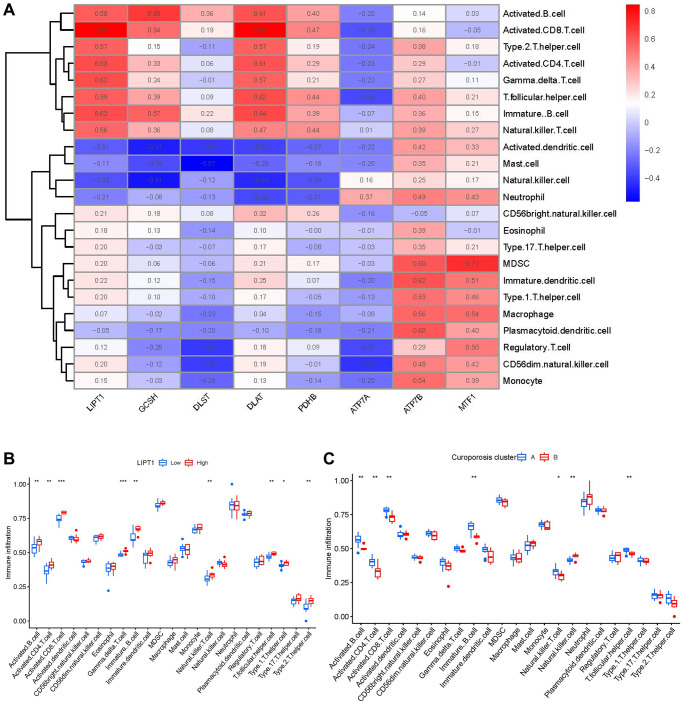
**Single sample gene set enrichment analysis.** (**A**) Correlation between immune cell infiltration and the 8 significant cuproptosis modulators. (**B**) Difference in the abundance of infiltrating immune cells between high and low LIPT1 protein expression groups. (**C**) Differential immune cell infiltration between clusterA and clusterB. ^*^p < 0.05, ^**^p < 0.01, and ^***^p < 0.001.

Then, we investigated the links between immune cells and 8 important cuproptosis regulators using ssGSEA to determine the immune cell abundance in IDD samples. We noticed a positive correlation between LIPT1 and many immune cells (Figure 7A). Between patients with high and low LIPT1 expressions, we examined the variations in immune cell infiltration. The findings revealed that patients with high LIPT1 expression intended to have a higher enhanced immune cell infiltration than patients with low LIPT1 expression (Figure 7B). We discovered that clusterA was correlated with activated B cell, activated CD8 T cell, activated CD4 T cell, immature B cell, natural killer T cell and T follicular helper cell immunity, while clusterB was linked to the immunity of natural killer cell (Figure 7C).

### Cuproptosis gene signature establishment through two distinct cuproptosis gene clusters

**Figure 8:**
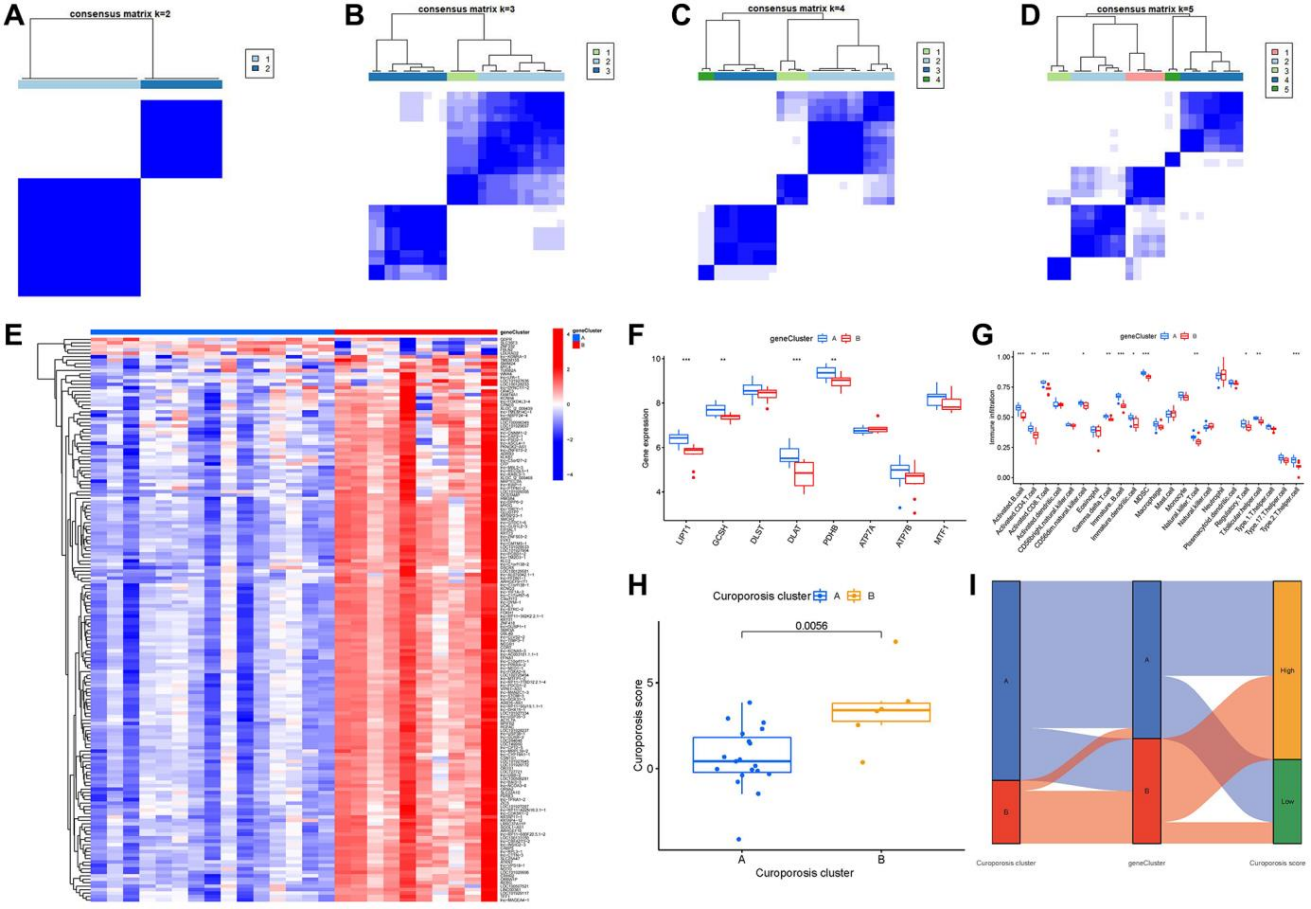
**Consensus clustering of the 163 cuproptosis-associated DEGs in IDD.** (**A**–**D**) Consensus matrices of the 163 cuproptosis-associated DEGs for k = 2–5. (**E**) Expression heat map of the 163 cuproptosis-associated DEGs in gene clusterA and gene clusterB. (**F**) Differential expression boxplot of the 8 significant cuproptosis modulators in gene clusterA and gene clusterB. (**G**) Differential immune cell infiltration between gene clusterA and gene clusterB. (**H**) Differences in cuproptosis score between clusterA and clusterB. (**I**) Sankey diagram showing the relationship between cuproptosis patterns, cuproptosis gene patterns, and cuproptosis scores. ^*^p < 0.05, ^**^p < 0.01, and ^***^p < 0.001.

On the basis of the 163 cuproptosis-associated DEGs, we classified the IDD cases into different genomic subtypes using a consensus clustering technique in order to illuminate the cuproptosis patterns. We discovered two unique cuproptosis gene patterns (gene clusterA and gene clusterB), which matched the sectionalization of cuproptosis patterns (Figure 8A–8D). The expression levels of the 163 cuproptosis-associated DEGs in gene clusterA and gene clusterB were shown in Figure 8E. The immune cell infiltration levels and the 8 important cuproptosis regulators between gene clusterA and gene clusterB were also analogous to those in the cuproptosis patterns (Figure 8F, 8G). The accuracy of our sectionalization by the consensus clustering method was once again confirmed by these results. PCA techniques were used to quantify the cuproptosis patterns by calculating the cuproptosis scores for each sample between the two different cuproptosis patterns. We discovered that the clusterB displayed higher cuproptosis score than clusterA (Figure 8H). Finally, we plotted a Sankey diagram to exhibit the relationships among cuproptosis scores, cuproptosis patterns, and cuproptosis gene patterns (Figure 8I).

### Identification of candidate compounds against IDD

**Figure 9:**
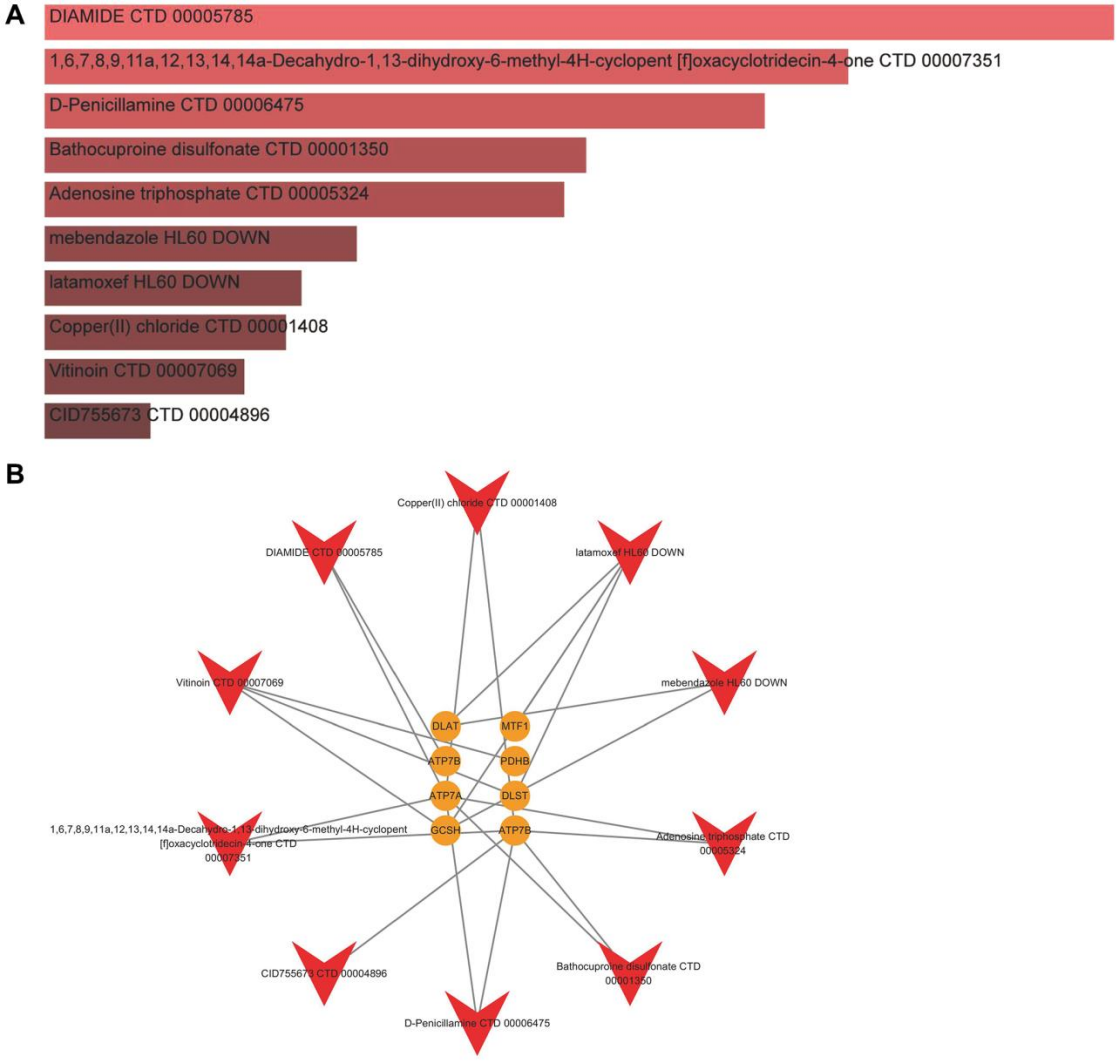
**Identification of candidate compounds against IDD.** (**A**) The top 10 effective compounds from the DSigDB database for 8 significant cuproptosis genes. (**B**) Compound-target network.

In the aspects of 8 significant cuproptosis genes as potential drug targets of IDD, we screened the top 10 compounds based on their *P*-value in the DSigDB database, as shown in Figure 9A and Supplementary Table 5. Then we visualized a compound-target network (Figure 9B) via Cytoscape (v3.7.2) software, which shows the relationships between effective compounds and 8 significant cuproptosis genes.

### Clinical sample validation

**Figure 10:**
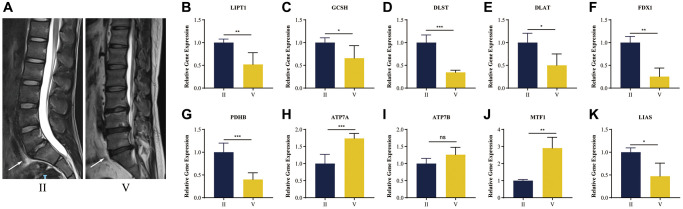
**RT-qPCR validation of human NP tissue.** (**A**) Typical human MR T2-weighted images of grade II and grade IV. The degenerated discs were indicated by white arrows. (**B**–**K**) Cuproptosis genes FDX1, LIAS, LIPT1, GCSH, DLST, DLAT, PDHB, exhibited significantly lower expression levels in IDD NP samples than controls, while ATP7A, ATP7B and MTF1 displayed the opposite results. All results were expressed as mean ± standard deviation. ^*^p < 0.05; ^**^p < 0.01; ^***^p < 0.001.

In order to understand the presence of cuproptosis during IDD, we collected NP tissue samples from patients. The T2-weight images of Magnetic Resonance exhibited degenerated discs of different degrees with varied signal intensity (Figure 10A).

It was verified that cuproptosis genes FDX1, LIAS, LIPT1, GCSH, DLST, DLAT, PDHB, exhibited significantly lower expression levels in IDD NP samples than controls, while ATP7A, ATP7B and MTF1 displayed the opposite results (Figure 10B–10K), which was consistent with the bioinformatics results.

### Histological staining analysis and RT-qRCR validation *in vivo*

**Figure 11:**
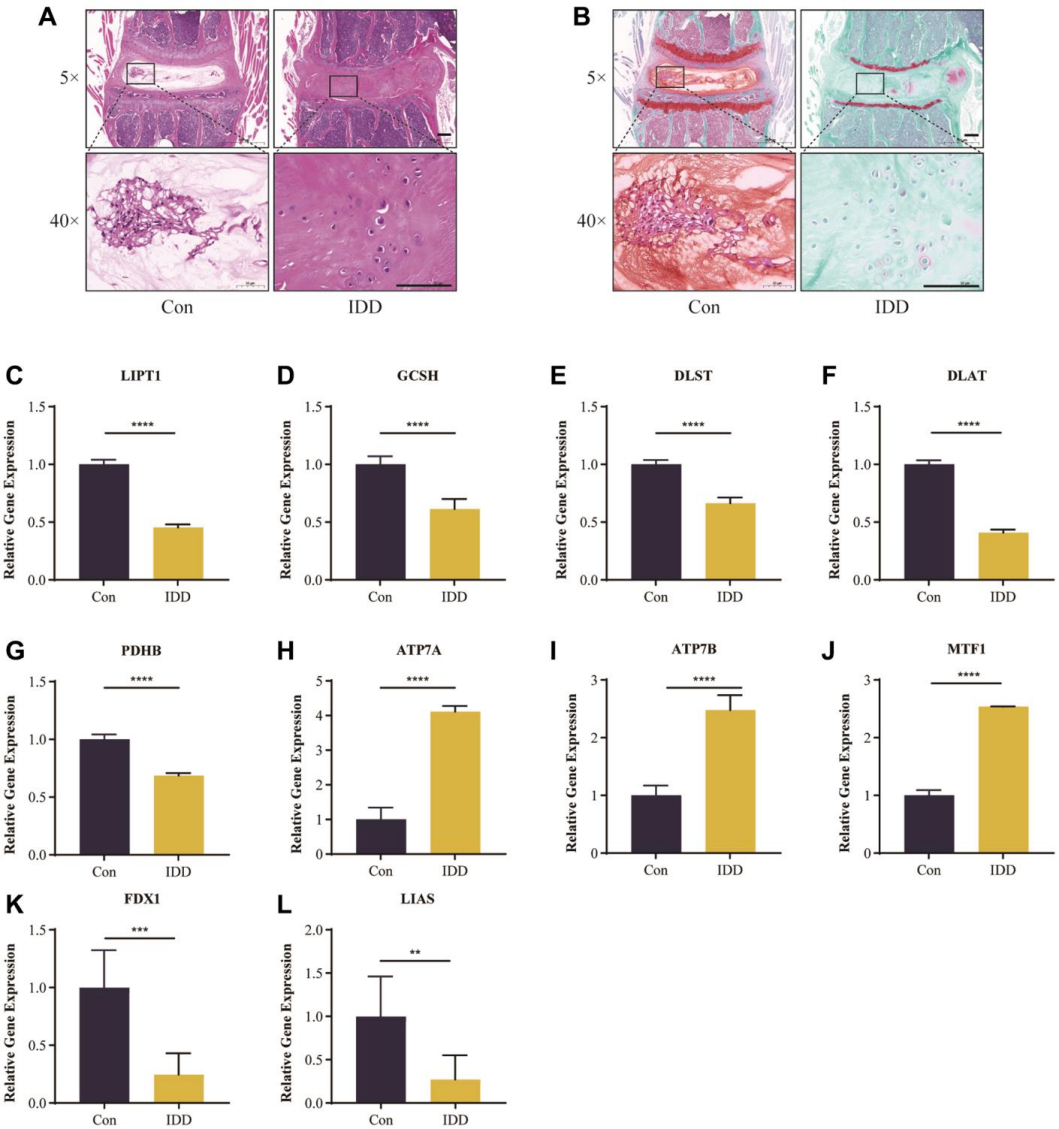
**Histological staining analysis and RT-qRCR validation *in vivo*.** Representative HE (**A**) and safranin O/green-stained (**B**) images of intervertebral discs in the control and IDD groups. (**C**–**L**) Cuproptosis genes FDX1, LIAS, LIPT1, GCSH, DLST, DLAT, and PDHB exhibited significantly lower expression levels in IDD NP samples than controls, while ATP7A, ATP7B and MTF1 displayed the opposite results. All results were expressed as mean ± standard deviation. ^**^p < 0.01; ^***^p < 0.001; ^****^p < 0.0001.

Figure 11A, 11B show the results of HE and safranin O/green staining. It is clear that the intervertebral discs in the IDD group exhibited greater degeneration compared to the control group, such as disappearance of the nucleus pulposus, which was replaced by disorganized annulus fibrosus; also, the orderly arrangement of the annulus fibrosus was destroyed and the endplate partly disappeared. This indicates successful establishment of an IDD model of naturally aging mice.

It was verified that cuproptosis genes FDX1, LIAS, LIPT1, GCSH, DLST, DLAT, PDHB, exhibited significantly lower expression levels in IDD NP samples than controls, while ATP7A, ATP7B and MTF1 displayed the opposite results (Figure 11C–11L), which was consistent with the bioinformatics results.

### Immunofluorescence analysis and RT-qRCR validation *in vitro*

**Figure 12:**
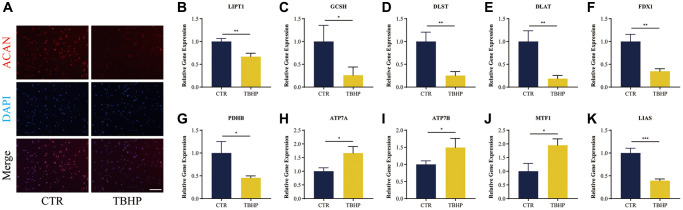
**Immunofluorescence analysis and RT-qRCR validation *in vitro*.** (**A**) Representative immunofluorescence images of ACAN in NP cells photographed by fluorescence microscopy; Scale bar = 100 μm. (**B**–**K**) Cuproptosis genes FDX1, LIAS, LIPT1, GCSH, DLST, DLAT, and PDHB exhibited significantly lower expression levels in IDD NP cell samples than controls, while ATP7A, ATP7B and MTF1 displayed the opposite results. All results were expressed as mean ± standard deviation. ^*^p < 0.05; ^**^p < 0.01; ^***^p < 0.001. Abbreviations: CTR: control; TBHP: tert-butyl hydrogen peroxide.

The assay concentrations of TBHP were 0, 50, 100, 150, 200 and 250 μmol/L (μM). The THBP concentration at 100 μM after 4 h suppressed the proliferation of NP cells (Supplementary Figure 1); in turn, this induced inflammation and oxidative stress, thereby contributing to the pathogenesis of IDD as reported previously [32, 33]. Thus, a THBP concentration of 100 μM (after 4 h) was selected for subsequent experiments. Importantly, NP-specific anabolic gene ACAN, have important functions in the pathological process of IDD [34]. Therefore, we also detected the protein expression of ACAN using immunofluorescence assays. As Figure 12A shows, fluorescence intensity of ACAN was reduced after TBHP treatment, which demonstrated the successful establishment of IDD cell model *in vitro*.

It was verified that cuproptosis genes FDX1, LIAS, LIPT1, GCSH, DLST, DLAT, PDHB, exhibited significantly lower expression levels in IDD NP cells indued by TBHP than controls, while ATP7A, ATP7B and MTF1 displayed the opposite results (Figure 12B–12K), which was consistent with the bioinformatics results.

## DISCUSSION

IDD results from the structure and function disorders of intervertebral discs, which impairs the maintenance of spinal stability causing low back pain, increasing a major financial and social burden throughout the world [35, 36]. In addition, the clinical treatment of IDD doesn’t focus on its pathogenesis but the symptoms, which can’t prevent its vicious circle process fundamentally [36]. Gene targeted therapies can delay or even reverse IDD at the genetic level by suppressing inflammatory cell death and inflammatory response, which may be a therapeutic strategy for IDD [37]. Cuproptosis is a copper-triggered manner of mitochondrial cell death [38]. Mounting evidence has revealed that copper metabolism plays an important role in multiple biological processes of IDD [14, 39]. It has been verified that copper complexes exert a toxic and immunogenic effect on intervertebral discs [15], suggesting that cuproptosis may exist in the pathological process of IDD. However, the role of cuproptosis modulators in IDD stays unclear due to scanty research. Our present study has revealed that these vital cuproptosis modulators in IDD show close correlations with cuproptosis-related mechanisms, which are mainly enriched in biological process (tricarboxylic acid metabolic process), cellular component (mitochondrial matrix), and molecular function (ATPase activity), while their KEGG enrichment analysis showed that Citrate cycle (TCA cycle) was one of the mainly enriched pathways (Figure 2J, 2K).

Firstly, a total of 8 significant cuproptosis modulators were screened from 16 cuproptosis modulators via differential expression analysis between controls and IDD cases, which were selected as diagnostic cuproptosis modulators (LIPT1, DLAT, PDHB, GCSH, DLST, ATP7A, ATP7B and MTF1) based on an established RF model to predict the occurrence of IDD. Then, we set up a nomogram model on the basis of the five candidate cuproptosis modulators (ATP7B, DLAT, MTF1, PDHB, GCSH), which has been evaluated via the DCA curve to produce benefit to IDD patients in virtue of decisions on the basis of the nomogram model.

Lipoic acid genes encoding components of the lipoic acid pathway are critical modulators of cuproptosis, and the synthesis of lipoic acid occurs in mitochondria under the catalysis of LIPT1, DLAT, LIAS and PDHB [7]. Lipoic acid can not only act synergistically with different types of heavy metals (such as copper, iron, mercury) to protect organs and tissues, but also accelerate the glucose metabolic cycle in mitochondria, enhancing the body’s metabolism and immune function [40, 41]. Lipoic acid could reverse mitochondrial apoptosis and damage caused by high glucose in cartilage endplate cells [42], and the presence of lipoic acid also affect human intervertebral disc tissue and extracellular matrix structure *in vitro* [43]. LIPT1 (lipolytransferase 1) is an enzyme activating 2-ketoacid dehydrogenases related to the TCA cycle [44], which has been reported to exert amidotransferase activity in disorders of human lipoate metabolism [45]. Protein targets of lipoylation are comprised of the pyruvate dehydrogenase (PDH) complex, including PDHB (pyruvate dehydrogenase E1 subunit beta) and DLAT (dihydrolipoamide S-acetyltransferase) [7, 46]. DLAT and PDHB are indispensable for immune microenvironmental conditions, which are closely correlated with carbohydrate metabolism and mitochondrial TCA cycle [47, 48]. Many studies have shown that the deficiency of PDHB leads to mitochondrial dysfunction and promotes glycolysis [49]. Existing study has confirmed that annulus fibrosus tissue highly expresses PDH, the enzyme responsible for lactate oxidative phosphorylation in the TCA cycle, which is evidence of lactate-dependent metabolic symbiosis in intervertebral discs [50]. These lipoylation targets and FDX1 are the key regulators of cuproptosis, when FDX1 knockout resulted in complete loss of protein lipoylation and triggered cuproptosis [7]. In our *in vivo* and *in vitro* experiments as well as clinical sample validation, we found that FDX1, LIAS, LIPT1, DLAT and PDHB were expressed at significantly lower level in IDD samples. It is suggested that lower expression of FDX1, LIAS, LIPT1, DLAT and PDHB may be involved in impacting lipoate metabolism and mitochondrial TCA cycle and triggering cuproptosis, which could contribute to the progression of IDD.

GCSH (glycine cleavage system protein H) and DLST (dihydrolipoamide S-succinyltransferase) serve as important enzymes activating a highly conserved lysine posttranslational modification called protein lipoylation, which are involved in metabolic complexes that regulate the entry of carbon into the TCA cycle [51, 52]. Existing evidence has confirmed that GCSH has a dual role in maintaining protein lipoylation and cell metabolism [53]. DLST has been reported to be the most importantly elevated gene in myeloid cells, and inhibition of DLST reduces expression and function of immunosuppressive markers as well as oxidative phosphorylation in myeloid cells [54]. In addition, numerous studies have confirmed that GCSH and DLST targeting glycine and glutamine metabolism have the potential to delay IDD through antioxidant effects [55–57]. In our *in vivo* and *in vitro* experiments as well as clinical sample validation, we found that GCSH and DLST were expressed at significantly lower level in IDD samples. It is suggested that lower expression of GCSH and DLST may be involved in impacting glycine and glutamine metabolism as well as antioxidant effects and thus triggering cuproptosis, which could contribute to the progression of IDD.

The mechanisms of cuproptosis involve the gene models of copper homeostasis dysregulation, including cuproptosis regulator MTF1 (metal-regulatory transcription factor 1), the copper exporters ATP7A (ATPase copper-transporting alpha) and ATP7B (ATPase copper-transporting beta), which are encoded by genes that are mutated in the copper dysregulation syndrome Wilson’s disease [58, 59]. The overexpression of ATP7A or ATP7B can lead to intracellular copper accumulation and cell death with aging-related diseases and various cancers [60, 61]. Through ATP hydrolysis, ATP7A and ATP7B are able to transport copper from the cytoplasm to the cell membrane, which have emerged as predictive markers for resistance to chemotherapy [62]. Existing report has shown that the ATPase-related subunits could enhance the anti-proliferative effect in the osmoregulatory response of nucleus pulposus cells [63]. It has been reported that the nuclear translocation of MTF1 could influence the progression of IDD *in vivo* [64]. In our *in vivo* and *in vitro* experiments as well as clinical sample validation, we found that ATP7A, ATP7B and MTF1 were expressed at significantly higher level in IDD samples. It is suggested that higher expression of ATP7A, ATP7B and MTF1 may be involved in copper accumulation and cell death, which could contribute to the progression of IDD.

Previous studies have shown that the number and phenotype of immune cell subpopulations in intervertebral disc tissues are closely correlated with the development and progression of IDD patients [65]. Abundant immune cells are infiltrated in the immune microenvironment of the intervertebral disc, triggering the inflammatory response of IDD, so it is considered to be a potential strategy to ameliorate IDD symptoms through immunotherapy targeting immune cells [66]. Our study demonstrated that the patients in clusterB with high-risk cuproptosis scores had a greater abundance of natural killer cell. Existing research shows that the activated natural killer cells take part in the pathological process of IDD [67]. Further study has confirmed that natural killer cells exert cytotoxic effects on nucleus pulposus cells [68]. However, the patients in clusterA with low-risk cuproptosis scores was related to activated CD4 T cell, activated B cell, etc. The adaptive immune response to IDD could be characterized by the activated subsets of T and B cells, and abnormal differentiation of T cells could trigger the overexpression of inflammatory molecules and abnormal expression of B cells, which has a significant connection with IDD [65, 69]. CD4 T cell subset has been reported to participate in regulating inflammatory response and provide immunomodulatory treatments for IDD [70]. B cells were activated to manufacture antibodies, which are involved in inflammation and immune responses of IDD [65]. Therefore, immunotherapy based on clusterA or clusterB would provide a promising therapeutic strategy against IDD.

In addition, our present study offers 10 candidate drugs against IDD from the DSigDB database for 8 significant cuproptosis genes based on the assessment of target-compound interactions, which is important to understand the structural features recommended for receptor sensitivity [27]. However, there exist some limitations to this study. Further experimental validations are necessary to confirm the specific mechanisms by which cuproptosis-related genes affect specific pathways and the immune microenvironment in IDD. To the best of our acknowledge, there exist no studies on the direct correlation between these candidate cuproptosis modulators and IDD. We expect that our present study can provide strategies for future experimental research on these cuproptosis modulators.

## CONCLUSION

In general, our present study screened 8 prognostic cuproptosis modulators and constructed a nomogram model providing accurate prediction for the prevalence of IDD. Then, we authenticated two cuproptosis patterns based on the 8 cuproptosis modulators. Importantly, we collected clinical IDD samples and successfully established IDD model *in vivo* and *in vitro*, and then carried out RT-qPCR experiments, indicating that cuproptosis genes FDX1, LIAS, LIPT1, GCSH, DLST, DLAT, PDHB, exhibited significantly lower expression levels in IDD samples than controls, while ATP7A, ATP7B and MTF1 displayed the opposite results, which was consistent with the bioinformatics results. To our knowledge, this study is the first to report cuproptosis-related prognostic biomarkers of IDD in the subtype classification and immune microenvironment infiltration.

## Supplementary Materials

Supplementary Figure 1

Supplementary Tables
