# Supplementary material for: Unravelling diagnostic clusters and immune landscapes of cuproptosis patterns in intervertebral disc degeneration through dry and wet experiments
Source: Aging (Albany NY). 2023 Dec 29;15(24):15599–623. doi: 10.18632/aging.205449 (PMC10781477; doi:10.18632/aging.205449)
Supplement: Supplementary Figure 1 [file aging-15-205449-s001.pdf]

SUPPLEMENTARY FIGURE

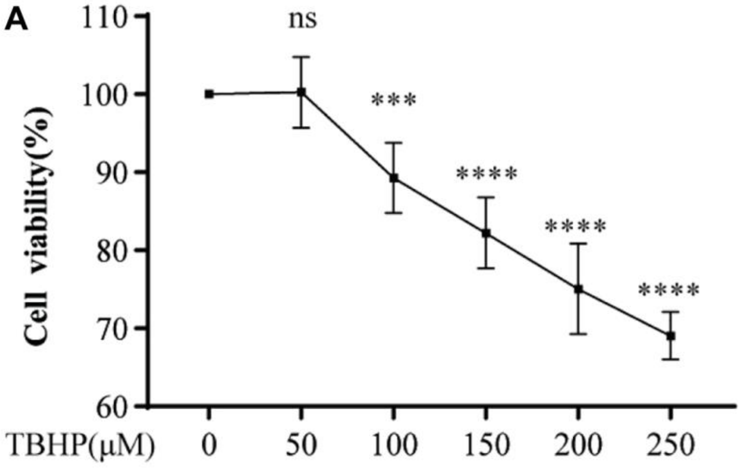

Supplementary Figure 1. CCK-8 assay. Cell viability was analyzed for NP cells treated with TBHP (concentration gradient = 0, 50, 100, 150, 200, and 250 μM) for 2, 4, and 6 h (**A**, **B**). Abbreviation: ns: non-significant, $^{***}p < 0.001$, $^{****}p < 0.0001$.
