# Supplementary material for: Unravelling diagnostic clusters and immune landscapes of cuproptosis patterns in intervertebral disc degeneration through dry and wet experiments
Source: Aging (Albany NY). 2023 Dec 29;15(24):15599–623. doi: 10.18632/aging.205449 (PMC10781477; doi:10.18632/aging.205449)
Supplement: Supplementary Tables [file aging-15-205449-s002.pdf]

SUPPLEMENTARY TABLES

Supplementary Table 1. Sequences of cuproptosis gene-specific primers used for RT-qPCR.

cuproptosis genes	Sequence (5′→3′)	
	Forward primer	Reverse primer
mmu-LIPT1	TGCTTCCGATTACTTTGTCAGC	TCCAGTCTTCAAAAGCCAGATTT
mmu-GCSH	ATGTCGCTGCAAGTGTCGAG	ACCGTTCCAATACCTTCCTCT
mmu-DLST	GGAACTGCCCTCTAGGGAGA	GACGCTACCACTGTTAATGACC
mmu-DLAT	CTTTAGCCTCCAAAGCGAGAG	AGATTGTAAATGTTCCACCCTGG
mmu-PDHB	AGGAGGGAATTGAATGTGAGGT	ACTGGCTTCTATGGCTTCGAT
mmu-ATP7A	ATGGAGCCAAGTGTGGATGC	GGTTGCACTCTTTTCGTCTAGT
mmu-ATP7B	GGGGACGATGCCTGAACAG	GCCGGGCAAAGCAAGTTTAG
mmu-MTF1	ACACCTTCGTCTGTAATCAGGA	CTGCACGTCACACTCAAATGG
mmu-FDX1	CAAGGGGAAAATTGGCGACTC	TTGGTCAGACAAACTTGGCAG
mmu-LIAS	CCTGGGGTCCCGGATATTTG	GAAGGTCTGGTCCATTATGCAA
hsa-LIPT1	CCTCTGTTGTAATTGGTAGGCAT	CTGGGGTTGGACAGCATTCAG
hsa-GCSH	GGAAGCGTTGGGAGATGTTGT	TCTGAAGGGTTACTCAGTGTCA
hsa-DLST	GAACTGCCCTCTAGGGAGAC	AACCTTCCTGCTGTTAGGGTA
hsa-DLAT	CGGAACTCCACGAGTGACC	CCCCGCCATACCCTGTAGT
hsa-PDHB	GAAGAGGCGCTTTCACTGGA	CAGCCCTCGACTAACCTTGT
hsa-ATP7A	TGACCCTAAACTACAGACTCCAA	CGCCGTAACAGTCAGAAACAA
hsa-ATP7B	GCCAGCATTGCAGAAGGAAAG	TGATAAGTGATGACGGCCTCT
hsa-MTF1	CACAGTCCAGACAACAACATCA	GCACCAGTCCGTTTTTATCCAC
hsa-FDX1	TTCAACCTGTCACCTCATCTTTG	TGCCAGATCGAGCATGTCATT
hsa-LIAS	CAGCCCAGTCAGACCGTTAAG	TTTCTGGCGTTTTAGGTTTCCT

Supplementary Table 2. The detailed information of GO enrichment analysis in Figure 2J.

Ontology	ID	Description	*p*-value	*p*.adjust
BP	GO:0006099	tricarboxylic acid cycle	3.07E-07	3.10E-05
BP	GO:0006101	citrate metabolic process	3.36E-07	3.10E-05
BP	GO:0006084	acetyl-CoA metabolic process	4.33E-07	3.10E-05
BP	GO:0072350	tricarboxylic acid metabolic process	4.69E-07	3.10E-05
BP	GO:0009060	aerobic respiration	5.38E-06	0.000285233
BP	GO:0018065	protein-cofactor linkage	7.22E-06	0.000314143
BP	GO:0006637	acyl-CoA metabolic process	9.48E-06	0.000314143
CC	GO:1990204	oxidoreductase complex	6.79E-08	2.17E-06
CC	GO:0005759	mitochondrial matrix	3.94E-07	6.30E-06
CC	GO:0016323	basolateral plasma membrane	0.003231767	0.023815441
CC	GO:0005802	trans-Golgi network	0.003809144	0.023815441
CC	GO:0045240	dihydrolipoyl dehydrogenase complex	0.004455238	0.023815441
CC	GO:0005770	late endosome	0.004465395	0.023815441
CC	GO:0045239	tricarboxylic acid cycle enzyme complex	0.005667284	0.025907585
MF	GO:0016417	S-acyltransferase activity	8.81E-05	0.000866396
MF	GO:0015662	ATPase activity, coupled to transmembrane movement of	9.38E-05	0.000866396

		ions, phosphorylative mechanism		
MF	GO:0016620	oxidoreductase activity, acting on the aldehyde or oxo group of donors, NAD or NADP as acceptor	0.000105606	0.000866396
MF	GO:0046915	transition metal ion transmembrane transporter activity	0.000111793	0.000866396
MF	GO:0016903	oxidoreductase activity, acting on the aldehyde or oxo group of donors	0.000159983	0.000898486
MF	GO:0016746	transferase activity, transferring acyl groups	0.0001739	0.000898486
MF	GO:0005507	copper ion binding	0.00030204	0.001207755

Supplementary Table 3. The detailed information of KEGG enrichment analysis in Figure 2K.

ID	Description	*p*-value	*p*.adjust
hsa01200	Carbon metabolism	1.27E-06	1.17E-05
hsa00020	Citrate cycle (TCA cycle)	1.55E-06	1.17E-05
hsa00620	Pyruvate metabolism	0.00067	0.00335
hsa04978	Mineral absorption	0.00109	0.00403
hsa00010	Glycolysis / Gluconeogenesis	0.00136	0.00403
hsa01524	Platinum drug resistance	0.00161	0.00403

Supplementary Table 4. The detailed information of GO enrichment analysis in Figure 6G.

Ontology	ID	Description	*p*-value
BP	GO:0030903	notochord development	0.000963804
BP	GO:0010288	response to lead ion	0.001932265
BP	GO:0060142	regulation of syncytium formation by plasma membrane fusion	0.002444549
BP	GO:0010038	response to metal ion	0.003384556
BP	GO:0010039	response to iron ion	0.003424517
CC	GO:0005882	intermediate filament	0.000457017
CC	GO:0045111	intermediate filament cytoskeleton	0.000997498
CC	GO:0005791	rough endoplasmic reticulum	0.001858512
CC	GO:0008076	voltage-gated potassium channel complex	0.023029928
CC	GO:0034705	potassium channel complex	0.028726385
MF	GO:0005184	neuropeptide hormone activity	0.003629045
MF	GO:0005200	structural constituent of cytoskeleton	0.003636338
MF	GO:0019869	chloride channel inhibitor activity	0.029550255
MF	GO:0005249	voltage-gated potassium channel activity	0.029806881
MF	GO:0097493	structural molecule activity conferring elasticity	0.032457692

Supplementary Table 5. The top 10 effective compounds from the DSigDB database for 8 significant cuproptosis genes.

Index	Compound name	*P*-value	Combined score	Targeted genes
1	DIAMIDE CTD 00005785	2.39E-05	4168.651313	ATP7B; ATP7A
2	1,6,7,8,9,11a,12,13,14,14a-Decahydro-1,13-dihydroxy-6-methyl-4H-cyclopent [f]oxacyclotridecin-4-one CTD 00007351	1.03E-04	1650.515154	ATP7B; ATP7A
3	D-Penicillamine CTD 00006475	1.63E-04	1233.639334	ATP7B; ATP7A
4	Bathocuproine disulfonate CTD 00001350	4.36E-04	658.5990148	ATP7B; ATP7A
5	Adenosine triphosphate CTD 00005324	4.92E-04	609.0948614	ATP7B; ATP7A
6	mebendazole HL60 DOWN	0.001538943	287.5086153	GCSH; DLAT

7	latamoxef HL60 DOWN	0.002086277	72.22540191	GCSH; MTF1; DLST; DLAT
8	Copper (II) chloride CTD 00001408	0.002272749	220.8403193	ATP7B; ATP7A
9	Vitinoin CTD 00007069	0.002856297	86.9234533	GCSH; DLST; PDHB
10	CID755673 CTD 00004896	0.004790703	1385.975083	ATP7B
